# Hidradenitis Suppurativa in Kuala Lumpur, Malaysia: A 7-Year Retrospective Review

**DOI:** 10.1155/2018/2017959

**Published:** 2018-05-22

**Authors:** Moonyza Akmal Ahmad Kamil, Azura Mohd Affandi

**Affiliations:** Department of Dermatology, Kuala Lumpur Hospital, Kuala Lumpur, Malaysia

## Abstract

**Introduction:**

Hidradenitis Suppurativa (HS) is a chronic inflammatory skin condition characterized by inflamed nodules, abscesses, sinus tracts, and scarring, which can occur in any skin containing folliculopilosebaceous units. We aim to identify the demographic and clinical characteristics and treatment modalities in patients with HS.

**Methods:**

A retrospective analysis involving records of patients diagnosed with HS in Hospital Kuala Lumpur from July 2009 to June 2016.

**Results:**

Sixty-two patients were identified, with equal cases involving males and females. Majority of patients were Malays (41.9%), followed by Indians (35.5%), Chinese (17.7%), and other ethnicities (4.8%). Median age at diagnosis was 25 (IQR: 14) years. There is a delay in diagnosis with a median of 24 (IQR: 52) months. Most of the patients had lesions on the axilla (85.5%), followed by groin (33.9%) and gluteal region (29%). Gluteal lesions were more common in males. Nodules (67.7%), sinuses (56.5%), and abscesses (33.9%) were the main clinical features, with 43.5% classified under Hurley stage 2. There was no difference in terms of symptoms and types of lesions among different ethnicities and genders. Majority received systemic antibiotics, more than half had retinoid, and third of the patients had surgical intervention.

**Conclusions:**

A prompt recognition of HS is imperative, to screen for comorbidities and to initiate early treatment to reduce physical and psychological complications.

## 1. Introduction

Hidradenitis Suppurativa (HS), also known as Verneuil's disease, fox den disease, pyoderma fistulans significa (PFS), or acne inversa, has now been increasingly recognised to be due to a chronic inflammatory recurring skin condition that involves the follicular occlusion of the folliculopilosebaceous units (FPSUs) [1]. HS mainly affects the intertriginous skin areas of the axillary, groin, perianal, perineal, genital, and inframammary skin, as well as other areas of the skin which contains FPSUs. Follicular occlusion, follicular rupture, and the associated innate and adaptive immune dysregulation seem to be the essential events that lead to the development of HS. Bacterial infection and colonization are being regarded as secondary pathogenic factor that may worsen HS. Melnik and Plewig had proposed HS as an autoinflammatory disease characterized by dysregulation of gamma-secretase/Notch pathway [2]. Elevated levels of proinflammatory cytokines such as tumor necrosis factor- (TNF-) *α*, interleukin- (IL-) 1*β*, and IL-17 have been reported in HS lesions. Furthermore, there are also reports on genetic susceptibility, mechanical stresses on the skin, obesity, smoking, and hormonal factors which are closely related to HS [3].

The prevalence of HS is varied and not well described. It ranges from 0.05% to 6%, depending on how and where the data were collected [4, 5]. The most recent large population based study involving >48 million patients in United States found a much higher incidence of 11.4 per 100,000 population [6].

HS has a wide spectrum of clinical manifestation. It ranges from the relatively mild cases characterized by recurrent appearance of papules, pustules, and inflammatory nodules, to severe cases of deep fluctuant abscesses, draining sinuses, and keloidal scars. Although HS is not a life-threatening condition, the pain, foul odour, and disfigurement are associated with significant decrease in quality of life [7]. The diagnosis of HS is made clinically but may be misdiagnosed especially by clinicians who are not familiar with this disease. In case of doubt, a skin biopsy may aid in excluding other diagnoses.

Hurley staging is frequently used to classify patients into 3 disease severity groups:

Stage I: abscess formation (single or multiple) without sinus tracts and scaring

Stage II: recurrent abscesses with sinus tracts and scarring, single, or multiple widely separated lesions

Stage III: diffuse or almost diffuse involvement, or multiple interconnected sinus tracts and abscesses across the entire area

HS is notoriously difficult to treat as it has multiple presentation and runs an unpredictable course. Treatment is based on disease severity, presence of comorbidities, patients' tolerance and preferability to the treatment, treatment cost, and the availability of treatment options. Besides the usual topical antibiotics and antiseptics dressings, there are systemic antibiotics, retinoids, and hormonal therapy. Recent evidence has shown that adalimumab and infliximab, 2 different monoclonal antibodies against TNF-*α*, are effective in the treatment of moderate to severe HS. Adalimumab is the first Food and Drug Administration (FDA) approved treatment for moderate to severe HS in adults. The European Medicine Agency has also recently accepted HS as an indication for adalimumab. In 2015, the Board of the Italian Society of Dermatology and Venereology (SIDeMaST) has also given out guidelines on the role of TNF-*α* inhibitors in the management of HS [8]. Other trial drugs, such as IL-1 receptor antagonist (anakinra) and Human IgG kappa monoclonal antibody to IL-1*β* (canakinumab), have also shown improvement in HS [9, 10]. Above all, patient education and psychological support play an important role in managing HS.

This study aims to identify the demographic and clinical characteristics and treatment modalities in patients with HS in Hospital Kuala Lumpur (HKL). We also compare the clinical characteristics of HS patients of different ethnicity and gender group.

## 2. Materials and Methods

This was a retrospective study involving all patients who had been clinically diagnosed with Hidradenitis Suppurativa from 1 July 2009 to 30 June 2016 in Dermatology Clinic, Kuala Lumpur Hospital. Based on the electronic record from the clinic registration, all of the clinic notes of these patients with the diagnosis of HS were traced and reviewed by 3 doctors from the clinic who had been trained to collect the data. Information such as the demographic information, clinical characteristics, and treatment modalities were extracted from patients' clinic notes and transcribed into the data collection form.

The collected data were analyzed using SPSS 22.0. Descriptive data were performed. All categorical variables such as gender and ethnicity, clinical characteristics, and treatment modalities were summarized as numbers and percentage, while continuous variables such as current age, age at diagnosis, and duration of illness before diagnosis were calculated and expressed in median and interquartile range (IQR). Assumption of normality was based on Kolmogorov-Smirnov statistics, skewness, kurtosis, histograms, and Q-Q plots. The majority of our continuous variables were not normally distributed; therefore, nonparametric tests were used for further data analysis. The tests used were chi-square test for independence, Fisher's exact test, and Mann–Whitney *U* test. A 2-tailed *p* value of <0.05 is considered as statistically significant.

## 3. Results

Of the 47067 records of new cases registered in HKL Dermatology Clinic from 1 July 2009 to 30 June 2016, a total of 62 patients were identified to have the diagnosis of HS. This led to an incidence rate of 0.013 per 100,000 clinic population. An equal number of cases were seen between females and males. HS were seen in patients between 14 and 79 years old. The median age at diagnosis was 25 (IQR: 14) years. Male patients were noted to be diagnosed at an older age (median age: 33, IQR 16 years old) when compared to female patients (median age: 24, IQR: 12 years old, *p* = 0.006). There was no significant difference between both genders in the duration of illness before being diagnosed with HS (*p* = 0.101).

Table 1 shows the demographic characteristics of patients with HS according to gender, ethnicity, age at diagnosis, and duration of illness before diagnosis. HS was more frequently seen in Malay (41.9%), followed by Indian (35.5%), Chinese (17.7%), and other ethnicities (4.8%). There was a delay in making the diagnosis with median duration of illness prior to diagnosis of 24 (IQR: 52) months, with the longest duration of 30 years. There were only 2 patients with similar family history of HS. Nine patients were cigarette smokers. Median BMI of our patients was 34.0 (IQR 20.2), calculated with missing data of up to 90%.

Table 2 shows the clinical characteristics of our study cohort. Majority of the patients had lesions over the axilla (85.5%), followed by groin (33.9%) and gluteal region (29%). Male patients had significant lesions over the gluteal region (*p* = 0.002) (Table 5).

The types of lesions commonly seen were nodules (67.7%), sinuses (56.5%), pustules (37.1%), keloid scar (35.5%), and abscesses (33.9%). More than half of them (56.5%) reported symptoms of pain, while a third (37.1%) reported pruritus. There was no significant difference detected in the type of lesions, severity, and symptoms of HS, in between different ethnicities, as well as between male and female patients (Tables 5 and 6)

About 35.5% of patients had positive swab or tissue culture, which grew a mixture of Gram-positive and Gram-negative organisms (Table 3).

**Figure 1:**
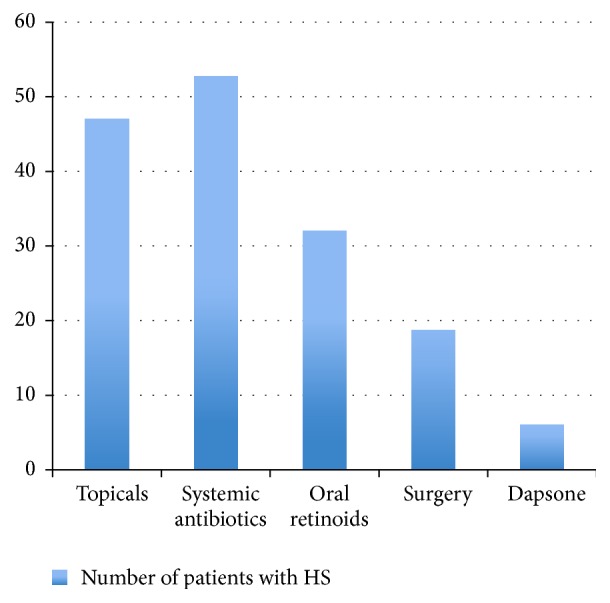
Treatment modalities of patients with HS.

In terms of treatment modalities, more than 75% of the patients had topical treatment, which include topical fusidic acid (30.6%), mupirocin (8.1%), clindamycin (4.8%), and antiseptic wash such as chlorhexidine, prontosan, and octenisan wash (66.1%). Fifty-two patients had received systemic antibiotics such as doxycycline (29%), rifampicin (29%), clindamycin (21.1%), tetracycline (8.1%), metronidazole (6.5%), and minocycline (1.6%). Thirty patients had isotretinoin while only 6 patients had acitretin. Six patients had dapsone for the treatment of HS. None of these patients had biologics. A third of these patients had some surgical intervention, which include incision and drainage (14 patients), localised excision (6 patients), and wide local excision and skin graft (3 patients).  Figure 1 shows the treatment modalities of patients with HS.

Table 4 shows the comorbidities of the patients with HS. Seventeen patients (27.4%) had Acne Conglobata or Nodulocystic Acne. Pilonidal sinus was diagnosed in 3 patients. None of these patients fulfilled the criteria of the follicular occlusion triad or tetrad, only 9 patients (14.5%) with obesity, 6 patients (9.7%) with diabetes mellitus, and 4 (6.5%) with hypertension and dyslipidemia. One patient had concurrent HIV and hepatitis C infection. Depression was also detected in 1 patient with HS. In Table 6, HS patients with concurrent hypertension and dyslipidemia are seen more in the Chinese population (*p* = 0.039, *p* = 0.031 consecutively).

## 4. Discussion

Sixty-two patients had the diagnosis of HS from the review of medical records from 1 July 2009 to 30 June 2016. This has shown an increasing trend when we compare to a similar study done by Leelavathi et al. which reported only 15 patients over 5-year period [11] (Table 7).

Many studies reported that HS is a rarely diagnosed disease [4, 12, 13]. Vazquez et al. reported that only 268 patients were diagnosed with HS in Minnesota between 1968 and 2008, with an overall annual age- and sex-adjusted incidence of 6.0 per 100,000. Cosmatos et al. found a low rate of clinically detected HS where the overall prevalence estimate was 0.053% in a retrospective analysis using health insurance database in the United States (Table 7). We found an incidence rate of 0.013 in 100,000 clinic population, which is much lower than previous studies. This is because Hospital Kuala Lumpur, being a fully subsidized government hospital, is a major tertiary center in Malaysia. Although primarily covering Kuala Lumpur and its surrounding area, it also has patients coming from other parts of the country, including almost the whole of Peninsular Malaysia.

We noted that the ratio of male to female patients was equal, which is inconsistent with previous studies [4, 5, 13–15]. Our number of male to female patients visits to dermatology clinic during this study period was almost equal (1 : 0.94). Shali Alikhan et al. reported that women outnumbered men with HS by nearly 3 to 1, and Garg et al. reported that prevalence of female patients was more than 2-fold greater than in male patients. The only study that reported an obvious male predominant in HS was seen in a study in Tunisia [16].

In our study, the median age at diagnosis was 25 (IQR: 14) years. Male patients with HS were found to be significantly older at the time of diagnosis compared to female patients, with median age of 33, IQR 16 years old for males, compared to median age of 24, IQR 12 years old for females, with *p* = 0.006. This is in line with a study in Minnesota which found that the highest incidence of HS is among young women aged 20–29 (18.4 per 100,000) [4]. Cosmatos et al. [13] found that most patients with HS were aged 30 to 64 years (Table 7).

The diagnosis of HS is often delayed. This is due to the fact that HS has a wide spectrum of manifestation and may mimic other illnesses and commonly treated as skin infections such as recurrent furuncles. This delay may be caused by patient delay in seeking treatment, the clinician not making the correct diagnosis, or both. The longest duration of diagnosis after the onset of symptoms in this study was 30 years. This patient had gone to multiple primary care physicians who had failed to give the accurate diagnosis. We also had a patient who had underwent several surgical interventions to the abscesses and only later diagnosed with HS. This finding is in line with previous studies, which reported significant delay in the diagnosis of HS [4, 17].

It was also surprising to see that, in our study, the proportion of Indians diagnosed with HS was much higher (35.5%) when compared with the number of new cases of Indian patients during the study period (18.1%). A study by McMillan in 2014 reported that the percentage of visits with HS diagnosis in black patients was significantly higher than the percentage of all visits by black patients in a combined data from the National Ambulatory Medical Care Survey (NAMCS) and National Hospital Ambulatory Medical Care Survey (NHAMCS) in the United States [12]. In another study, the adjusted HS prevalence among African American patients with HS was more than 3-fold than that among white patients [15]. An increased frequency of HS is observed in blacks, possibly because blacks have a greater density of apocrine glands than whites. This same explanation can be postulated in our finding associating HS with the Indian ethnicity in Malaysia, who has darker Fitzpatrick phototype as compared to other ethnicities such as Malay and Chinese.

Although there are emerging data on the association of obesity, metabolic syndrome, and other related comorbidities with HS [18–20], we did not find significant difference statistically between obesity and metabolic syndrome with HS. This is due to the limited documentation in our retrospective study. Missing data were up to 90% in certain categories, as they were not documented in patients' record.

However, with the limited data, we noted that Chinese patients had significantly higher percentage of having concurrent hypertension and dyslipidemia, but there was no difference of comorbidities in between males and females. With the combination of smoking in patients with HS, these contribute to a higher risk of cardiovascular-associated death, as shown in Danish HS patients [21]. None of our patients reported having inflammatory bowel disease or rheumatology condition, as described previously. Previous studies have found that patients with HS have a higher risk of developing depression [22]. In our study, only 1 patient had been diagnosed with depression and currently being treated by the psychiatry team. The low prevalence of depression may be due to the fact that psychiatry illness is still a taboo in our country and therefore patients do not openly express their emotional distress to the clinician.

Majority of the patients had lesions over the axilla, followed by groin and gluteal region, inframammary, chest, back, neck, and postauricular region. The lesions were composed of nodules, sinuses, pustules, keloid scar, and abscesses. At diagnosis, majority of the patients had moderate to severe symptoms (Hurley stages 2 and 3) and more than half of them reported symptoms of pain, while a third reported pruritus. Previous studies had reported that the distribution of lesions in HS is influence by gender. Primary sites of involvement in females are the groin or upper inner thigh, axilla, upper anterior torso (including breast and inframammary regions), and the buttocks or gluteal clefts [4, 23]. In males, the common sites are the groin or thigh, axilla, perineal or perianal regions, and buttocks or gluteal cleft [24]. However, when comparing between males and females in our patients with HS, there was no significant difference between the severity, symptoms of HS, or the location of lesions, except that male patients had more significant lesions over the gluteal region.

Although routine bacterial culture is not indicated, we noted that patients with secondary bacterial infection worsened HS condition. In this study, the swab and/or tissue culture grew mixture of Gram-positive and Gram-negative organism, which can aid in the choice of antibiotics for these patients.

The most common oral tetracycline regime in our clinic is doxycycline, given in the highest percentage of patients, at the dosage of 100 mg once to twice daily. This is the key treatment for mild to moderate HS with favourable adverse effect profile. Combination therapy with clindamycin and rifampicin is the next option, usually to those who have failed oral tetracycline. Our regimen of combination therapy is clindamycin 300 mg twice daily and rifampicin 300 mg twice daily. Previous studies have shown that 35%–70% of patients had at least some improvement in their disease course, with sustained efficacy of more than 40% in 1 year [25–27].

Besides systemic antibiotics, more than half of our cohort received oral retinoid. Most of these patients were on isotretinoin, especially those with concurrent nodulocystic acne. Studies involving acitretin treatment have reported significant improvements in up to 60% of patients [27]. It is also more effective when used as an adjuvant to other systemic medications. Surgical interventions were also treatment choice for those with recurrent and complicated HS.

Although recent studies have shown that monoclonal antibodies against TNF-*α* are effective in moderate to severe HS, none of our patients had yet to receive biologic treatment [28]. This is due to our limited financial resources in this fully funded government clinic. Perhaps when the generic version of biologics or “biosimilars” is widely available, and cost is not an issue anymore, more patients would be on biologic treatment especially those with recalcitrant HS.

We had difficulty in ascertaining the outcome of treatment, as most of them had received multiple treatment modalities in their course of management. Furthermore, we noted that about half of these patients had defaulted on their appointment. This is the limitation that needs to be addressed as this study relied on previous documentations on patients' medical record. In addition, our result may have limited generalizability, as the population size is very small. Therefore, a larger, prospective cohort study is recommended to look specifically at the treatment modalities and the outcome of each treatment in the near future.

## 5. Conclusion

In conclusion, there is an equal gender distribution in our HS patients, with higher proportion in Indian population. Most of our patients were young adults, with a median age of diagnosis of 25 years, with long delay in the diagnosis of HS. Thus, it is pertinent that clinicians should be able to make an accurate diagnosis with comprehensive clinical examination, to screen for the associated comorbidities and to initiate treatment early to avoid the physical and emotional complications of this debilitating chronic skin disease.

## Figures and Tables

**Table 1:** Demographic characteristics of patients with HS.

Characteristics	*n* (%)	Median (IQR)
Gender		
Male	31 (50)	
Female	31 (50)	
Ethnicity		
Malay	26 (41.9)	
Indian	22 (35.5)	
Chinese	11 (17.7)	
Others	3 (4.8)	
Current age (years old)		31.5 (14)
Age at diagnosis (years old)		25.0 (14)
Duration of illness before diagnosis (months)		24.0 (52)

**Table 2:** Clinical characteristics of patients with HS.

Characteristics	*n* (%)
Types of lesions	
Nodules	42 (67.7)
Sinus	35 (56.5)
Pustules	23 (37.1)
Keloid scar	22 (35.5)
Abscess	21 (33.9)
Double comedones	2 (3.2)
Fistula	2 (3.2)
Location	
Axilla	53 (85.5)
Groin	21 (33.9)
Gluteal	18 (29)
Chest	10 (16.1)
Back	9 (14.5)
Neck	8 (12.9)
Inframammary	4 (6.5)
Genitalia	2 (3.2)
Postauricular	1 (1.6)
Hurley staging	
1	22 (35.5)
2	27 (43.5)
3	13 (21)
Symptoms	
Pain	35 (56.5)
Pruritus	23 (37.1)
Embarrassment	12 (19.4)

**Table 3:** Cultures taken from patients with HS.

Bacteriological culture (swab or tissue)	Number (%)
Gram-positive	
*Staphylococcus*	7 (11.3)
MSSA	4 (6.5)
MRSA	3 (4.8)
GBS	4 (6.5)
*Streptococcus pyogenes*	1 (1.6)
Diphtheroids	4 (6.5)
*Enterococcus*	1 (1.6)
Gram-negative	
*E. coli*	6 (9.7)
*Proteus* sp.	5 (8.1)
*Klebsiella pneumonia*	4 (6.5)
*Acinetobacter*	2 (3.2)
Mixed growth	7 (11.3)
Others	
*Chrysosporium* sp.	1 (1.6)

MSSA: Methicillin Sensitive *Staphylococcus aureus*, MRSA: Methicillin Resistant *Staphylococcus aureus*, and GBS: Group B *Streptococcus*.

**Table 4:** Comorbidities in patients with HS.

Comorbidities	Number (%)
Acne conglobata/nodulocystic	17 (27.4)
Obesity	9 (14.5)
Diabetes	6 (9.7)
Hypertension	4 (6.5)
Dyslipidemia	4 (6.5)
Pilonidal sinus	3 (4.8)
Anemia	3 (4.8)
Neoplasm (SCC)	1 (1.6)
RVD and hepatitis C	1 (1.6)
Depression	1 (1.6)

**Table 5:** Comparison of HS clinical characteristics between male and female patients with HS.

Clinical characteristics	Male (*n* = 31) *n* (%)	Female (*n* = 31) *n* (%)	*p* value
*Severity of HS*			
Mild (Hurley stage 1)	8 (25.8)	14 (45.2)	0.111
Moderate to severe (Hurley stage 2-3)	23 (74.2)	17 (54.8)	
*Symptoms *			
Embarrassment	4 (12.9)	0 (0)	0.113
Pain	18 (58.1)	17 (54.8)	0.798
Pruritus	11 (35.5)	12 (38.7)	0.793
*Location of lesion*			
Axilla	25 (80.6)	28 (90.3)	0.279
Groin	9 (29.0)	12 (38.7)	0.421
Gluteal	15 (48.4)	3 (9.7)	0.002
Chest	4 (12.9)	6 (19.4)	0.490
Back	7 (22.6)	2 (6.5)	0.071
Neck	2 (6.5)	6 (19.4)	0.130
Inframammary	2 (6.5)	2 (6.5)	1.000
Genitalia	1 (3.2)	1 (3.2)	1.000
Postauricular	1 (3.2)	0 (0)	0.313
*Types of lesion*			
Papules	8 (25.8)	7 (22.6)	0.767
Nodules	22 (71.0)	20 (64.5)	0.587
Sinus	19 (61.3)	16 (51.6)	0.442
Pustules	12 (38.7)	11 (35.5)	0.793
Keloid scar	13 (41.9)	9 (29.0)	0.288
Abscess	12 (38.7)	9 (29.0)	0.421
Double comedones	1 (3.2)	1 (3.2)	1.000
Fistula	1 (3.2)	1 (3.2)	1.000
*Comorbidities*			
Nodulocystic acne	9 (29.0)	8 (25.8)	0.776
Obesity	4 (12.9)	5 (16.1)	0.718
Diabetes	4 (12.9)	2 (6.5)	0.671
Hypertension	3 (9.7)	1 (3.2)	0.612
Dyslipidemia	2 (6.5)	2 (6.5)	1.000

**Table 6:** Comparison of clinical characteristics of HS among different ethnicity group.

Clinical characteristics	Malay (*n* = 26) *n* (%)	Chinese (*n* = 11) *n* (%)	Indian (*n* = 22) *n* (%)	Others (*n* = 3) *n* (%)	Total (*n* = 62)	*p* value
*Severity of HS*						
Mild	9 (34.6)	3 (27.3)	9 (40.9)	1 (33.3)	22 (35.5)	0.892
Moderate-severe	17 (65.4)	8 (72.7)	13 (59.1)	2 (66.7)	40 (64.5)	
*Symptoms *						
Pain	14 (53.8)	4 (36.4)	15 (68.2)	2 (66.7)	35 (56.5)	0.357
Pruritus	8 (30.8)	3 (27.3)	10 (45.5)	2 (66.7)	23 (37.1)	0.443
Embarrassment	1 (3.8)	1 (9.1)	1 (4.5)	1 (33.3)	12 (19.4)	0.246
*Location of lesion*						
Axilla	23 (88.5)	6 (54.5)	21 (95.5)	3 (100)	53 (85.5)	0.022
Groin	7 (26.9)	6 (54.5)	7 (31.8)	1 (33.3)	21 (33.9)	0.440
Gluteal	6 (23.1)	7 (63.6)	5 (22.7)	0 (0)	18 (29)	0.061
Chest	5 (19.2)	0 (0)	5 (22.7)	0 (0)	10 (16.1)	0.310
Back	4 (15.4)	4 (36.4)	1 (4.5)	0 (0)	9 (14.5)	0.089
Neck	4 (15.4)	2 (18.2)	2 (9.1)	0 (0)	8 (12.9)	0.766
Inframammary	3 (11.5)	0 (0)	0 (0)	1 (33.3)	4 (6.5)	0.072
Genitalia	1 (3.8)	0 (0)	1 (4.5)	0 (0)	2 (3.2)	0.892
Postauricular	1 (3.8)	0 (0)	0 (0)	0 (0)	1 (1.6)	0.704
*Types of lesion*						
Nodules	18 (69.2)	8 (72.7)	15 (68.2)	1 (33.3)	42 (67.7)	0.620
Sinus	14 (53.8)	8 (72.7)	11 (50.0)	2 (66.7)	35 (56.5)	0.624
Pustules	6 (23.1)	6 (54.5)	9 (40.9)	2 (66.7)	23 (37.1)	0.180
Keloid scar	9 (34.6)	6 (54.5)	7 (31.8)	0 (0)	22 (35.5)	0.316
Abscess	7 (26.9)	4 (36.4)	8 (36.4)	2 (66.7)	21 (33.9)	0.553
Double comedones	0 (0)	0 (0)	1 (4.5)	1 (33.3)	2 (3.2)	0.083
Fistula	2 (7.7)	0 (0)	0 (0)	0 (0)	2 (3.2)	0.413
*Comorbidities*						
Acne conglobata/nodulocystic	8 (30.8)	3 (27.3)	6 (27.3)	0 (0)	17 (27.4)	0.734
Obesity	5 (19.2)	1 (9.1)	3 (13.6)	0 (0)	9 (14.5)	0.741
Diabetes	3 (11.5)	2 (18.2)	0 (0)	1 (33.3)	6 (9.7)	0.152
Hypertension	1 (3.8)	3 (27.3)	0 (0)	0 (0)	4 (6.5)	0.039
Dyslipidemia	1 (3.8)	2 (18.2)	0 (0)	1 (33.3)	4 (6.5)	0.031

**Table 7:** Comparison between our study cohort and previous studies.

Variables	Vazquez et al., 2013	Cosmatos et al., 2013	Vinding et al., 2014	Leelavathi et al., 2009	Our study
Country	Minnesota, USA	USA	Copenhagen, Denmark	HKL	HKL
Year	1968–2008	2007	2014	2002–2006	2009–2016
Study type	Retrospective, medical records	Registry, insurers database	Self-administered questionnaires	Retrospective, medical records	Retrospective, medical records
Number of cases	268 cases 6.0/100,000 (incidence)	0.053% (7927/144,000) (prevalence)	2.10%, (Total patients 16404) (prevalence)	15 cases	62 cases 0.013/100,000 hospital population (incidence)
Age at diagnosis	Median 30.6 years (9.9–78.5)	Mean 38.2 (14.73) years		Mean 27.8 years (21–30)	Median 25.0 (12–69) years
Gender F : M	2.2 : 1	3 : 1	1.6 : 1	2.8 : 1	1 : 1
Duration between onset of symptom & diagnosis	3.3 years (0 to 30 years)	-	-	-	24 months (1 to 360 months)
Obesity	54.9%		72.9%	13.3% (33% missing data)	9 patients (14.5%) (up to 90% missing data)

## Data Availability

The datasets generated during and/or analysis in this study is available from the corresponding author on reasonable request.
